# Importance of correct femoral tunnel placement to avoid MPFL graft failure: a case report of habitual patellar dislocation after proximal physis fixation

**DOI:** 10.3389/fped.2026.1889183

**Published:** 2026-10-13

**Authors:** Yi Hua, Wenfei Peng, Hua Wang, Xu Yang

**Affiliations:** 1 Department of Joint Surgery and Sports Medicine, Zhongnan Hospital of Wuhan University, Wuhan, China; 2 Department of Orthopedics, Xiantao First People’s Hospital, Xiantao, China; 3 Department of Sports Medicine, Wuhan Fourth Hospital, Wuhan, China; 4 Hubei Provincial Sports Medicine Center, Wuhan, China

**Keywords:** case reports, MPFL, femoral fixation, proximal to the physis, distal to the physis

## Abstract

Evidence from anatomical studies shows that the femoral origin of the medial patellofemoral ligament (MPFL) is situated distal to the epiphyseal line in children. This case report describes a 9-year-old girl who underwent reconstruction of the medial patellofemoral ligament (MPFL) following an initial patellar dislocation. Due to her open growth plates, the MPFL graft was fixed proximal to the epiphyseal line using bone tunnel fixation. Due to ongoing femoral growth, the femoral tunnel migrated significantly in a proximal direction over the four years following surgery, leading to graft failure and the onset of habitual patellar dislocation.

## Introduction

Habitual patellar dislocation (HPD) is a relatively uncommon type of patellar instability characterized by lateral patellar dislocation during knee flexion and spontaneous relocation with extension of the knee (1, 2). With its favourable outcomes and low complication rate, medial patellofemoral ligament (MPFL) reconstruction is now considered a cornerstone in the treatment of patellar dislocation (3, 4). However, challenges remain for MPFL reconstruction in skeletally immature patients, particularly regarding femoral fixation techniques. In MPFL reconstruction, the femoral-side bony fixation technique may carry a risk of physeal injury (5). While soft tissue fixation spares the physis, it fails to provide adequate fixation strength, which is associated with a higher rate of postoperative redislocation (6). Therefore, to avoid physeal injury while ensuring adequate fixation strength, the femoral tunnel was positioned distal to the epiphyseal line for MPFL reconstruction in skeletally immature patients (7). Some orthopedic surgeons believe that the femoral fixation site should be located proximal to the epiphyseal line (8–10). However, positioning the femoral fixation site proximal to the physis carries inevitable drawbacks. As the lower limb grows, the original position of the femoral tunnel may migrate proximally, and the tension and length of the graft will also change accordingly, leading to re-dislocation of the patella (9–11). We present a case of proximal migration of the femoral tunnel after MPFL reconstruction with femoral fixation proximal to the epiphyseal line, which resulted in HPD.

## Case report

The patient was diagnosed with patellar subluxation of the right knee at the age of 9 (weight 42 kg, height 150 cm) (Figure 1). The patient underwent MPFL reconstruction and lateral retinacular release under general anesthesia at local hospital. A diagnostic arthroscopy was performed. However, no obvious intra-articular lesions were found. The ipsilateral autograft gracilis tendon was harvested. The patellar fixation was performed at the midpoint of the patella using a bone tunnel, and the femoral fixation was placed proximal to the physis using a bone tunnel. Postoperative radiographs demonstrated good patellofemoral congruence, with the femoral fixation site positioned proximal to the physis (Figure 2). Routine quadriceps exercises and knee range of motion exercises were performed. At the 10-month postoperative follow-up, the patient had returned to sports. However, CT and radiographs indicated lateral patellar subluxation and proximal femoral tunnel migration (Figure 3). As the patient was entirely asymptomatic, revision surgery was not undertaken at that time. Based on the imaging findings, we hypothesize that the lower limb growth led to the tunnel migrating proximally, altering graft length and tension, which resulted in patellar subluxation. However, the patient did not undergo revision surgery.

**Figure 1:**
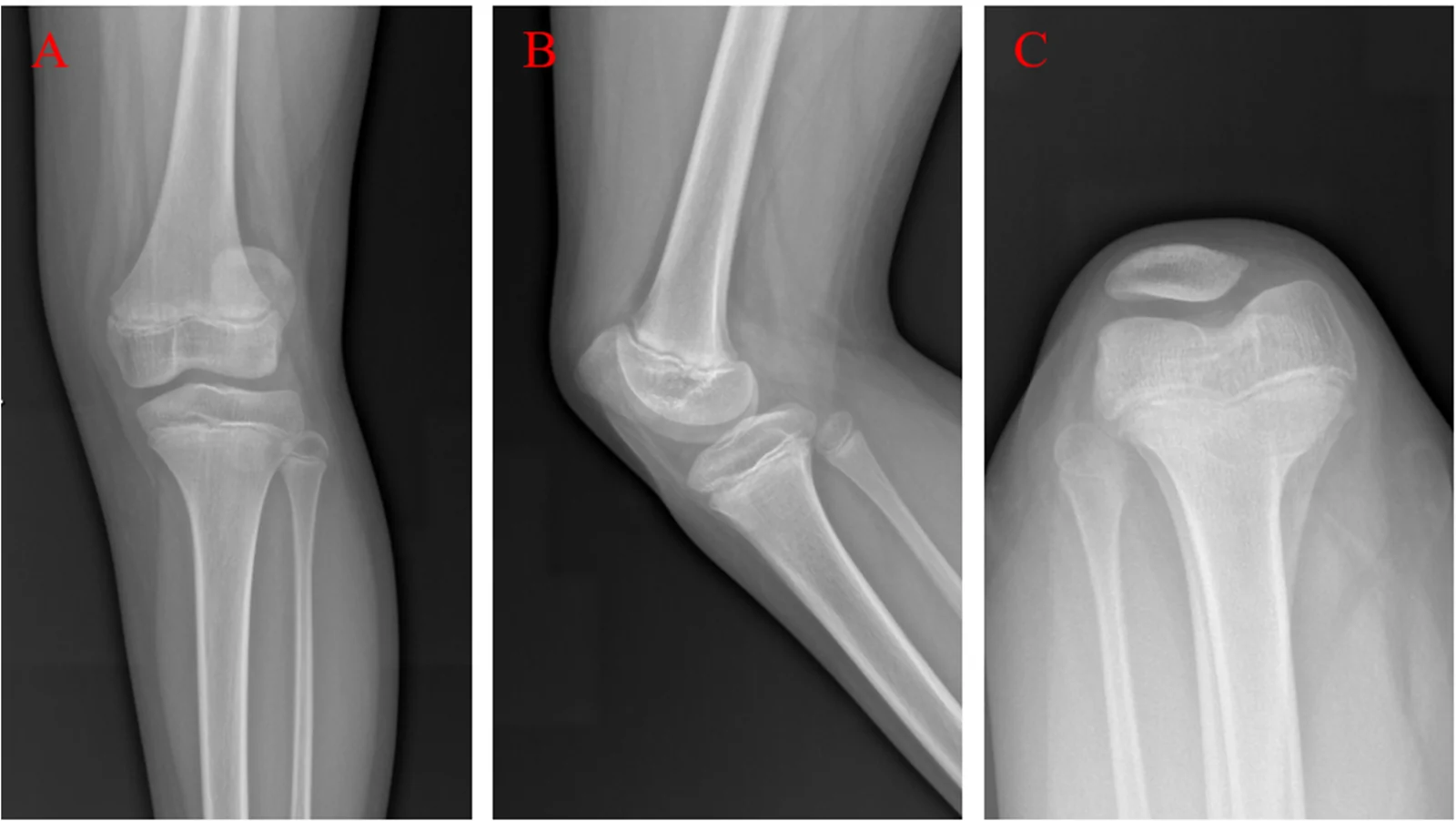
Preoperative Fluoroscopic view of the right knee at the age of 9.

**Figure 2:**
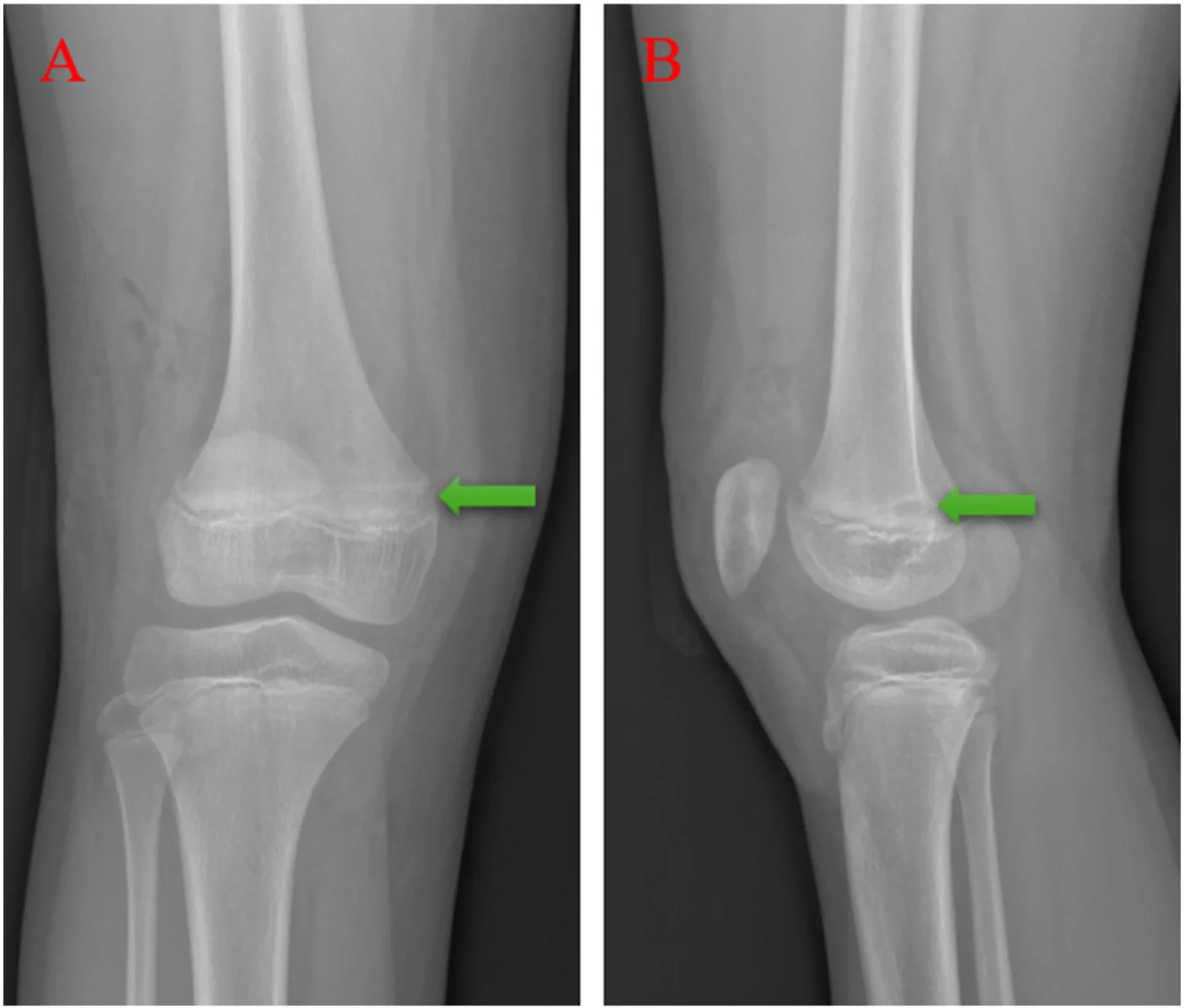
Postoperative radiographs of the right knee. The femoral tunnel was positioned proximal to the epiphyseal line (green arrow).

**Figure 3:**
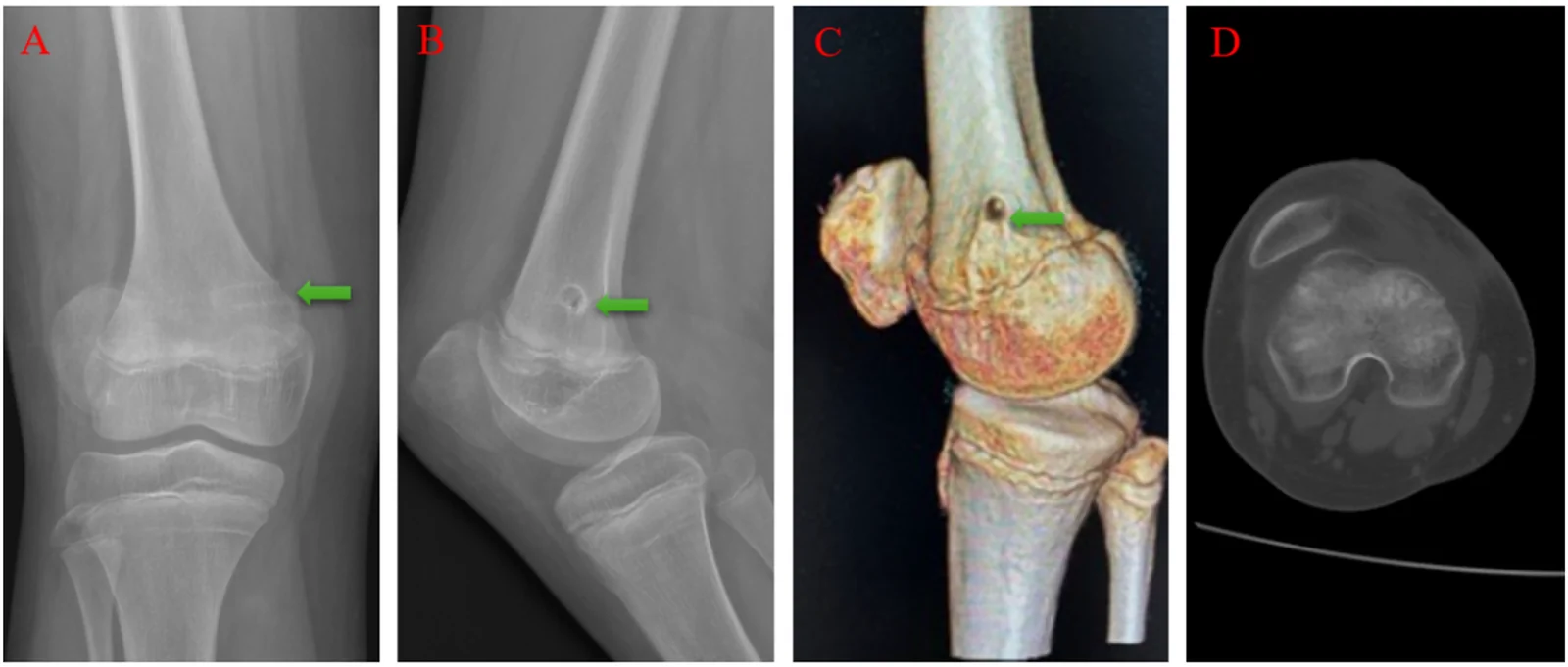
Radiographs and CT scans of the right knee at 10 months postoperatively (age 10) demonstrating proximal femoral tunnel migration (green arrows) and patellar subluxation.

Four years after the surgery, the patient experienced HPD in flexion of the right knee without apparent cause and sought treatment at our hospital. CT and radiographs of the right knee showed that the femoral tunnel migrated proximally (Figure 4). CT measurements demonstrated a femoral anteversion angle of 25.42° and a tibial tuberosity–trochlear groove (TT-TG) distance of 28.69 mm. (Figure 5).

**Figure 4:**
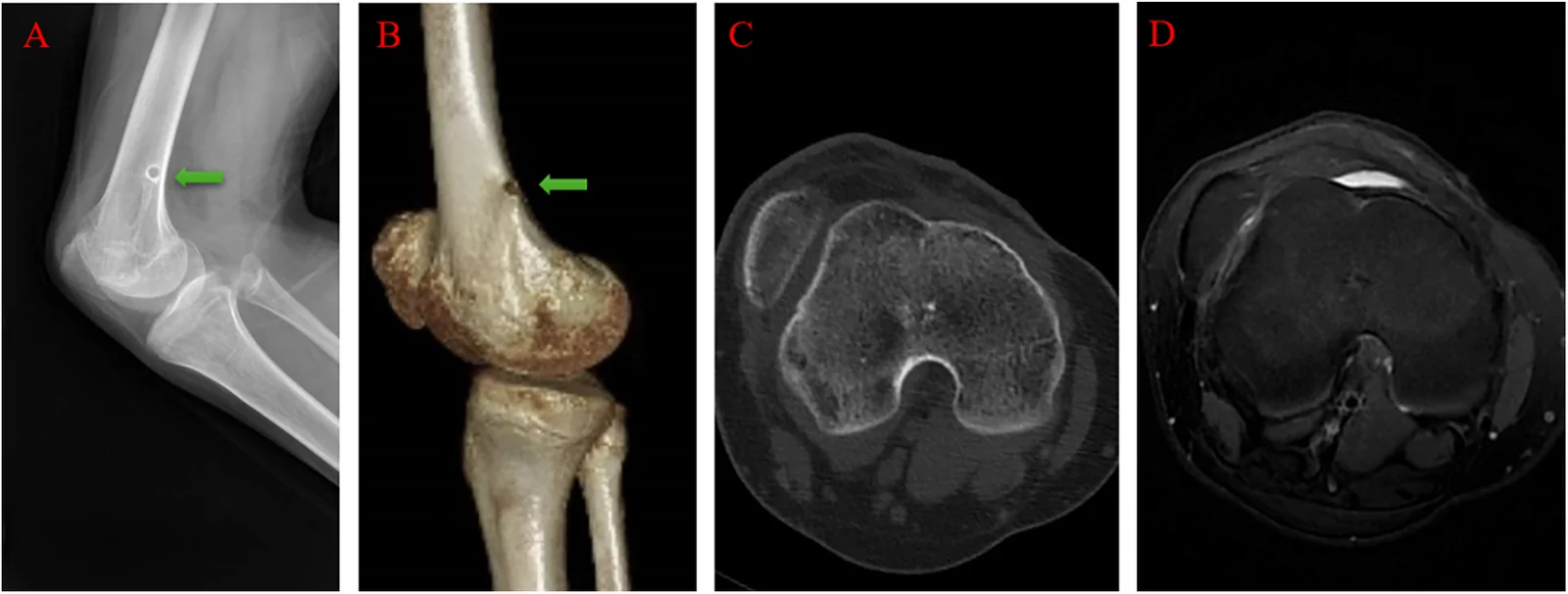
Radiographs, CT and MRI scans of the right knee at 4 years postoperatively (age 13). **(A,B)**. Significant proximal migration of the femoral tunnel (green arrows).

**Figure 5:**
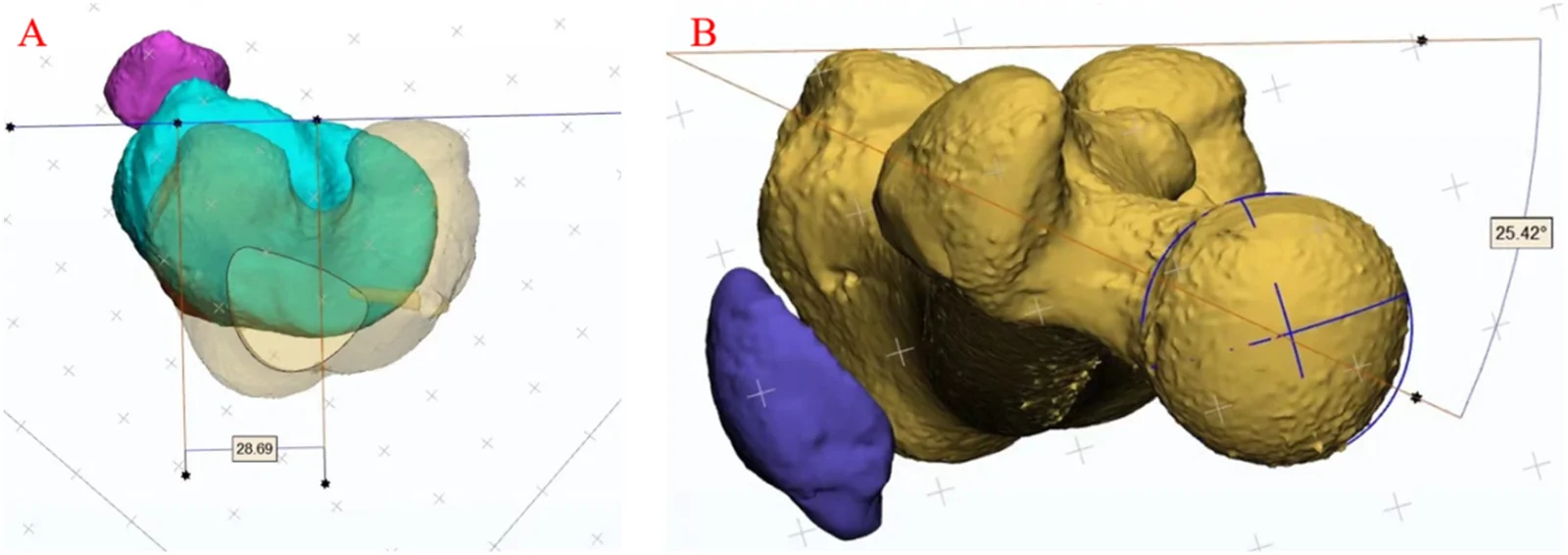
Preoperative measurement using 3D modelling of CT data of the right knee. **(A)** The TT-TG distance measured 28.69 mm. **(B)** The FAA measured 25.42°.

The first step of the revision surgery was to release the lateral contracted soft tissues of the knee. A stepwise lengthening of the extensor mechanism was performed, sequentially releasing the lateral retinaculum, iliotibial band, vastus lateralis tendon, and quadriceps tendon. A 10-cm longitudinal incision was made on the right knee. The lateral retinaculum of the right knee was incised in a Z-shape, with distal release extending to the lateral patellar tendon and proximal exposure of the vastus lateralis muscle. The patella still dislocated spontaneously upon knee flexion, so an L-shape release of the vastus lateralis tendon was performed. Following vastus lateralis tendon release, the patella remains dislocated during knee flexion, prompting an additional procedure involving separation between the rectus femoris and vastus intermedius muscle layers (Figure 6A).

**Figure 6:**
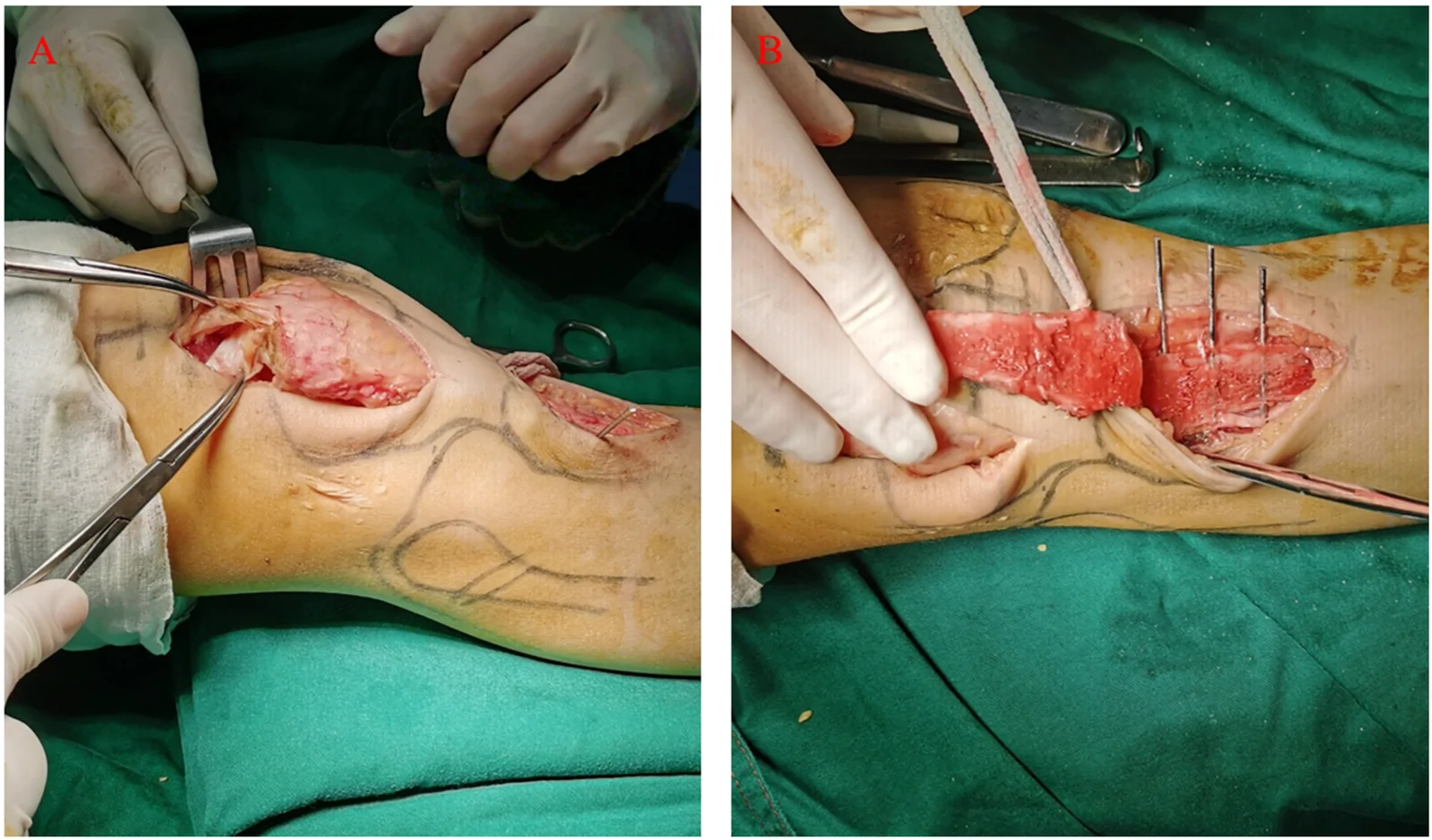
**(A)** separation of the deep and superficial layers of the quadriceps tendon. **(B)** Tibial tubercle osteotomy.

After the release of the contracture tissue, proximal and medial transfer of the tibial tubercle was performed (Figure 6B). A 10 cm longitudinal incision was made over the medial aspect of the tibial tuberosity to expose the tibial tubercle and patellar tendon. A semitendinosus tendon autograft was used. Then, the tibial tubercle osteotomy procedure was performed as described, creating a bone block 6 cm long and 2 cm wide. The bone block was subsequently translated medially by 10 mm and proximally by 8 mm, and then fixed with three 3.5 mm cortical bone screws.

MPFL reconstruction was performed after tibial tubercle osteotomy. Two tunnels were drilled in the midpoint and proximal third of the patella with a 2-mm K-wire. Two 4.5 mm suture anchors were placed, the graft was then fixed to the patella through the sutures of the two suture anchors. Under intraoperative fluoroscopic guidance, the femoral tunnel was positioned 7 mm distal to the epiphyseal line (12). It was then drilled to a depth of 30 mm using a 5-mm drill. Next, both ends of the graft were introduced into the femoral tunnel via a superficial capsular approach. With the knee flexed to 120°, the graft was fixed in the tunnel using a 6 mm interference screw. Full range of motion showed normal patellar tracking. The incision was closed in layers, and routine dressings were applied. Postoperative radiographs and CT scans of the knee showed good patellofemoral alignment (Figure 7).

**Figure 7:**
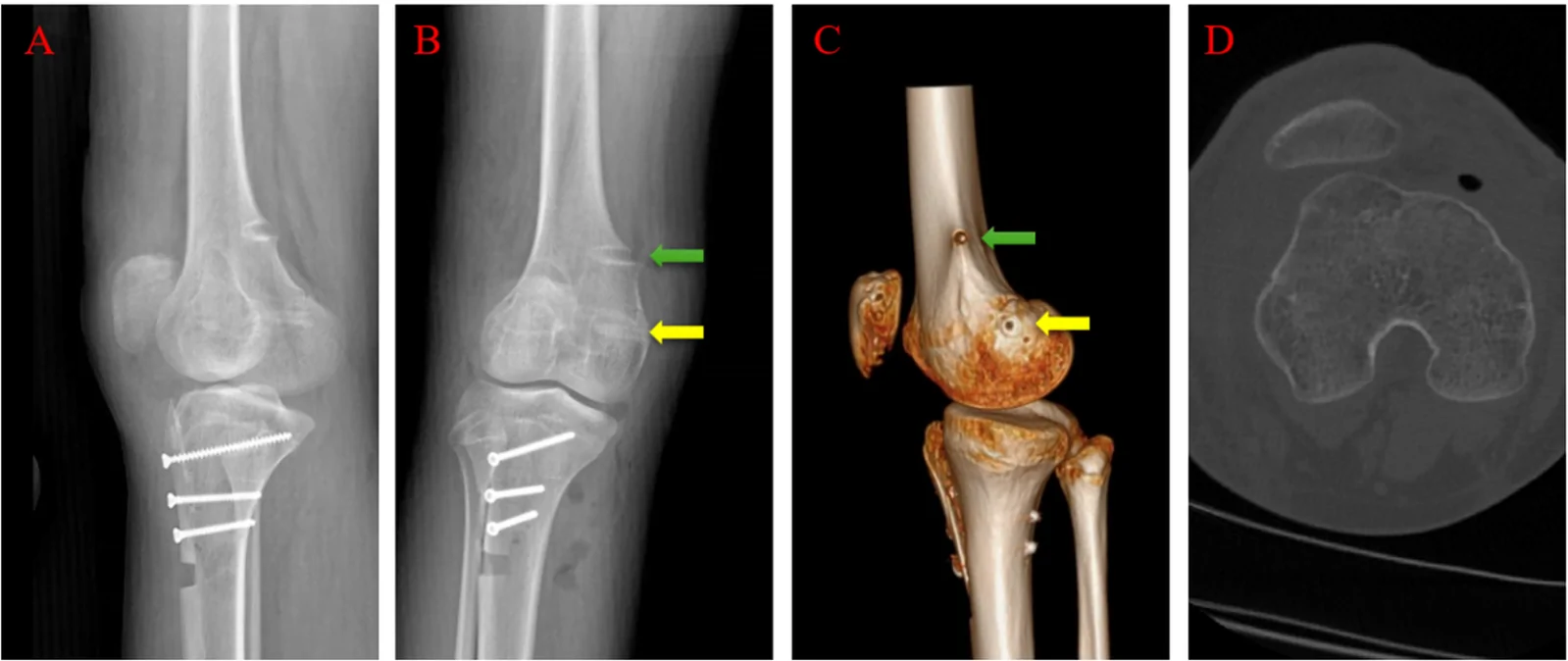
Radiographs and CT of the right knee postoperatively. Femoral tunnel from the first MPFL reconstruction (green arrows) and femoral tunnel position during the second MPFL reconstruction (yellow arrows).

At 1-year postoperative, the patient returned to our hospital for removal of the tibial tubercle fixation screws. By that time, the patient had returned to sports activities without complications such as infection or re-dislocation (Figures 8A,B). Intraoperative arthroscopic examination revealed well-aligned patellofemoral joint with no significant cartilage injury (Figure 8C).

**Figure 8:**
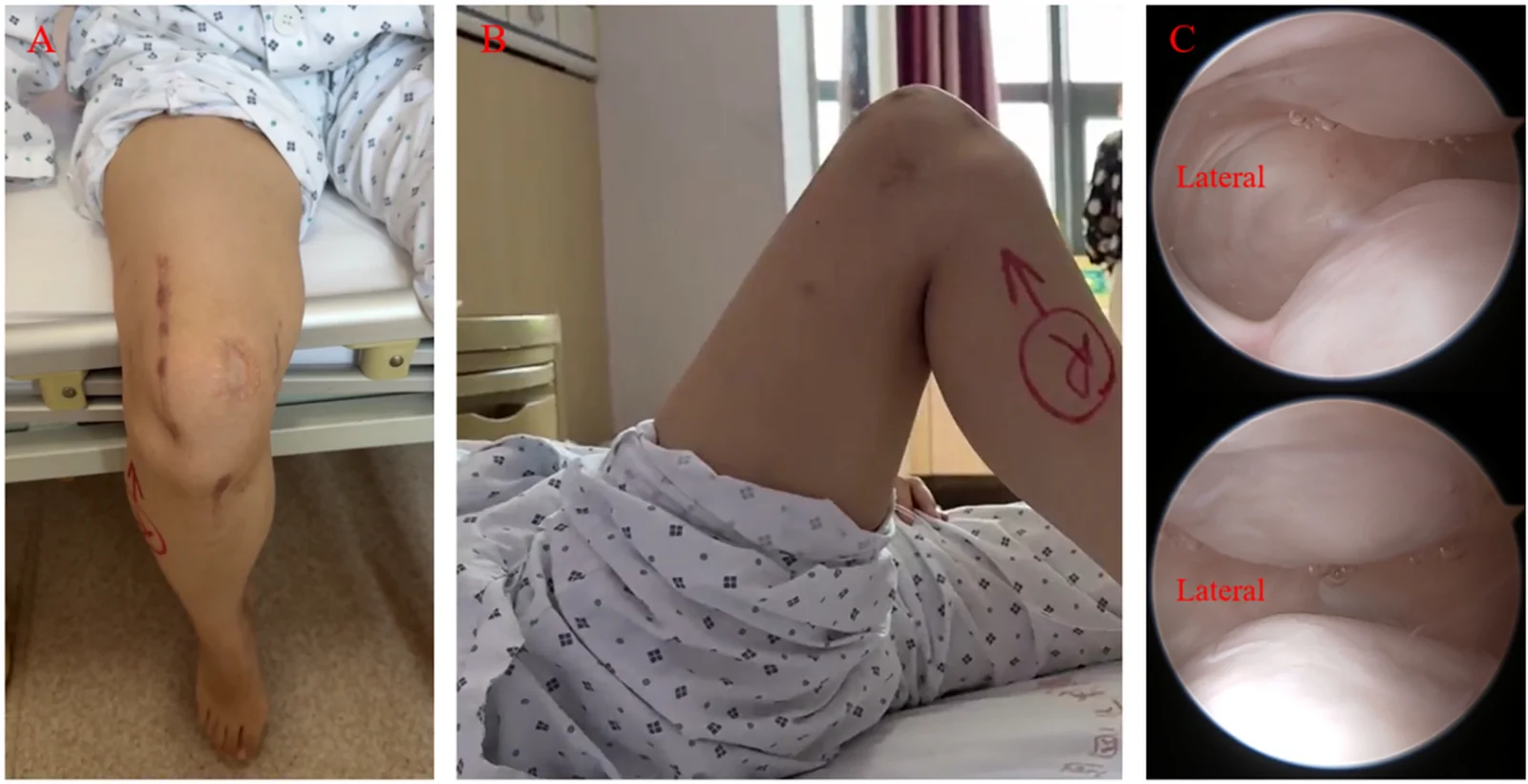
Follow-up 1 year after surgery. **(A)** Flexion of the knee at 90°. **(B)** Flexion of the knee at 120°. **(C)** Arthroscopic view of the patellofemoral joint.

## Discussion

We report a case of HPD caused by improper femoral tunnel selection during primary MPFL reconstruction in children. In this case, as the femoral tunnel migrates proximally, the graft is elongated, and the initial transverse stress acting on the patella gradually decreases, resulting in patellar subluxation.

The patient underwent MPFL reconstruction after the first patellar subluxation with the femoral tunnel intentionally placed proximal to the physis to avoid physeal injury. At 10 months postoperatively, radiographs and CT revealed patellar subluxation and the femoral tunnel had migrated proximally due to the femoral growth. Four years postoperatively, the patient experienced HPD due to graft failure from proximal tunnel migration.

To our knowledge, this is the first case reported in the literature of HPD caused by improper femoral tunnel selection during primary MPFL reconstruction in a skeletally immature patient.

Furthermore, a systematic literature search did not identify any previous case reports on this topic.

Using PubMed as an example, the search strategy was as follows: (“habitual patellar dislocation” OR “patellar dislocation”) AND (“MPFL reconstruction” OR “medial patellofemoral ligament reconstruction”) AND (“proximal physis” OR “physeal” OR “skeletally immature”) AND (“migration” OR “anchor migration” OR “tunnel migration”). MPFL reconstruction in skeletally immature patients poses challenges given the risk of physeal injury. Previously, MPFL reconstruction was typically performed using femoral soft tissue fixation to avoid physeal injury, such as medial collateral ligament pulley techniques (13, 14). However, soft tissue femoral fixation may be associated with poorer clinical outcomes, including a higher redislocation rate (6).

In this case, the femoral tunnel was positioned proximal to the epiphyseal line to avoid physeal injury during the first MPFL reconstruction. However, subsequent femoral growth resulted in significant proximal migration of the tunnel, ultimately causing graft failure and leading to HPD four years postoperatively. A femoral tunnel positioned proximal to the physis can increase patellofemoral joint contact stresses and excessive graft tensioning, which can result in graft failure and recurrent lateral instability (9, 15). Kupczik et al. (9) also reported proximal anchor migration in MPFL reconstruction due to the anchor being positioned proximal to the growth plate. Numerous anatomical and cadaveric studies have demonstrated that the femoral attachment of the MPFL is located distal to the epiphyseal line (16–18). Gruskay et al. (7) reported that the native MPFL attaches 4 to 9 mm distal to the epiphyseal line in children. Sochacki et al. (12) reported that the MPFL originates a mean distance of 7 mm distal to the epiphyseal line. Fixation of the graft distal to the epiphyseal line for MPFL reconstruction can respect the growth plates, leading to better graft isometry. In a biomechanical study, Black et al. (19) found that MPFL reconstruction with the femoral attachment site distal to the epiphyseal line was more isometric than those proximal to the epiphyseal line.

In a prospective study, Nelitz et al. (20) reported the outcomes of MPFL reconstruction with femoral tunnel placement distal to the physis for treating RPD with open growth plates. During a mean follow-up of 2.8 years, no re-dislocations or growth disturbances were observed, and the Kujala score significantly improved from 72.9 to 92.8. However, femoral fixation distal to the epiphyseal line does not entirely eliminate the risk of physeal injury. Seitlinger et al. (21) reported a case of physeal injury following MPFL reconstruction with distal epiphyseal fixation. This suggests that caution is warranted when performing such procedures in skeletally immature individuals, as interference with the growth plate can lead to undesirable effects on bone development. For skeletally immature patients undergoing MPFL reconstruction, continuous monitoring with regular clinical and radiographic evaluations is recommended until skeletal maturity.

## Conclusion

In conclusion, for MPFL reconstruction in pediatric, it is recommended that the femoral tunnel be placed distal to the epiphyseal line, as fixation proximal to the physis may lead to graft failure.

## Data Availability

The datasets presented in this article are not readily available because of ethical and privacy restrictions. Requests to access the datasets should be directed to the corresponding author.
